# Draft Genome Sequences of Two Xanthomonas vesicatoria Strains from the Balkan Peninsula

**DOI:** 10.1128/genomeA.01558-14

**Published:** 2015-02-12

**Authors:** Taca Vancheva, Nevena Bogatzevska, Penka Moncheva, Pierre Lefeuvre, Ralf Koebnik

**Affiliations:** a Faculty of Biology, Sofia University St. Kliment Ohridski, Sofia, Bulgaria; b UMR 186 IRD-Cirad-Université Montpellier 2 Résistance des Plantes aux Bioagresseurs, Montpellier, France; c Institute of Soil Science, Agrotechnologies, and Plant Protection Nikola Poushkarov, Sofia, Bulgaria; d CIRAD, UMR PVBMT, Pôle de Protection des Plantes, Saint-Pierre, Ile de la Réunion, France

## Abstract

Xanthomonas vesicatoria causes bacterial spot disease of pepper and tomato plants. We report here the first genome sequences of X. vesicatoria strains that have been isolated from pepper plants. These data will be used for comparative genomics and will allow the development of new detection and typing tools for epidemiological surveillance.

## GENOME ANNOUNCEMENT

Xanthomonas vesicatoria is one of the causal agents of bacterial spot disease on pepper and tomato plants (1). The disease symptoms include chlorotic and necrotic lesions on leaves, stems, petioles, fruits, and flowers, as well as defoliation. Bacterial spot disease is present worldwide where environmental conditions are suitable for the pathogen. The disease is one of the economically most important plant diseases on the Balkan Peninsula (2, 3), with annual losses reaching 10 to 20% (4). Bacterial spots of tomato were first recorded in Bulgaria in 1936 (5). Later, the disease was also reported on pepper plants in Bulgaria (6). The first report of bacterial spot disease in Macedonia dates back to 1999 (3). Current control of the disease is mainly based on agricultural practices and the use of copper compounds. In order to develop new molecular markers for epidemiological surveillance, such as variable-number tandem repeats (VNTR) (7), we sequenced the genomes of two strains from the Balkan Peninsula.

Strain 15b, isolated from Capsicum annuum in Sofia, Bulgaria, in 2005, and strain 53M, isolated from C. annuum in Strumitsa, Macedonia, in 2005, were chosen as representative X. vesicatoria strains from the Balkan Peninsula based on their pathogenic, physiologic, and genetic characteristics (8). Their genomes were sequenced using the Illumina HiSeq 2500 platform (Fasteris SA, Switzerland). The shotgun sequencing yielded 2,163,782 100-bp paired-end reads (541 Mb) for strain 15b and 2,007,779 paired-end reads (502 Mb) for strain 53M, with insert sizes ranging from 250 bp to 1.5 kb. The draft genome sequences were assembled using the Edena algorithm version 3.131028 (9), yielding 303 contigs of >500 bp (*N*_50_, 31,449 bp) for strain 15b and 320 contigs (*N*_50_, 29,340 bp) for strain 53M. The contigs were annotated with GeneMarkS+ release 2.9 (revision 452131) (10), as implemented in the NCBI Prokaryotic Genome Annotation Pipeline (http://www.ncbi.nlm.nih.gov/genome/annotation_prok/), which predicted a total of 4,601 genes for strain 15b and 4,624 genes for strain 53M.

Combined with the draft genome sequence of the New Zealand strain X. vesicatoria ATCC 35937, which was isolated from Lycopersicon lycopersicum (11), these genomic resources will aid in the development of new diagnostic tools, such as multilocus VNTR analyses (MLVA), which have been proven to be powerful tools for studying bacterial phytopathogen populations (12–15). In addition, these genome sequences may give new insight into the pathogen-host interaction. For instance, a comparison of the predicted repertoires of type III effectors with the effector repertoire of the tomato isolate X. vesicatoria ATCC 35937 revealed that both pepper isolates appear to lack *xopD*, *xopE2*, and *xopJ2*. Contrariwise, the pepper isolates possess homologs of *avrBs1* and *xopH*, which were not found in strain ATCC 35937. Both genes are in tandem arrangement and might be located on a plasmid, as in Xanthomonas euvesicatoria strain 85-10 (16). More sequencing and pathotyping are required to assess the importance of these effectors with respect to the host plants.

### Nucleotide sequence accession numbers. 

These whole-genome shotgun projects have been deposited at DDBJ/EMBL/GenBank under the accession numbers JSXZ00000000 (strain 15b) and JSYJ00000000 (strain 53M). The versions described in this paper are the first versions, JSXZ01000000 and JSYJ01000000.
